# High quality *de novo* sequencing and assembly of the *Saccharomyces arboricolus* genome

**DOI:** 10.1186/1471-2164-14-69

**Published:** 2013-01-31

**Authors:** Gianni Liti, Alex N Nguyen Ba, Martin Blythe, Carolin A Müller, Anders Bergström, Francisco A Cubillos, Felix Dafhnis-Calas, Shima Khoshraftar, Sunir Malla, Neel Mehta, Cheuk C Siow, Jonas Warringer, Alan M Moses, Edward J Louis, Conrad A Nieduszynski

**Affiliations:** 1 Institute of Research on Cancer and Ageing of Nice (IRCAN), CNRS UMR 7284 - INSERM U1081, Université de Nice Sophia Antipolis, 06107, NICE Cedex 2, France; 2 Department of Cell & Systems Biology, University of Toronto, M5S 2 J4, Toronto, Canada; 3 Centre for the Analysis of Genome Evolution and Function, University of Toronto, M5S 3B2, Toronto, Ontario, Canada; 4 DeepSeq, Centre for Genetics and Genomics, Queen’s Medical Centre, University of Nottingham, NG7 2UH, Nottingham, UK; 5 Centre for Genetics and Genomics, Queen’s Medical Centre, University of Nottingham, NG7 2UH, Nottingham, UK; 6 Current address: INRA, UMR1318, Institut Jean-Pierre Bourgin, F-78000, Versailles, France; 7 Department of Chemistry and Molecular Biology, University of Gothenburg, 41390, Gothenburg, Sweden

## Abstract

**Background:**

Comparative genomics is a formidable tool to identify functional elements throughout a genome. In the past ten years, studies in the budding yeast *Saccharomyces cerevisiae* and a set of closely related species have been instrumental in showing the benefit of analyzing patterns of sequence conservation. Increasing the number of closely related genome sequences makes the comparative genomics approach more powerful and accurate.

**Results:**

Here, we report the genome sequence and analysis of *Saccharomyces arboricolus*, a yeast species recently isolated in China, that is closely related to *S. cerevisiae*. We obtained high quality *de novo* sequence and assemblies using a combination of next generation sequencing technologies, established the phylogenetic position of this species and considered its phenotypic profile under multiple environmental conditions in the light of its gene content and phylogeny.

**Conclusions:**

We suggest that the genome of *S. arboricolus* will be useful in future comparative genomics analysis of the *Saccharomyces* sensu stricto yeasts.

## Background

The budding yeast, *Saccharomyces cerevisiae*, is a leading system in genomics due to the small genome size (12 Mb) and the availability of powerful genetic techniques. Genome sequencing of multiple *hemiascomycete* yeasts and multiple individuals from several species have allowed the application of a range of powerful comparative approaches. Comparative genomics have revealed evolutionary mechanisms that shape genomes and provided a formidable tool for assigning function to DNA sequence [1,2].

The closely related sensu stricto *Saccharomyces* species (*S. cerevisiae*, *S. paradoxus*, *S. mikatae*, *S. kudriavzevii*, *S. arboricolus* and *S. bayanus*) provide a clade with multiple genetically tractable species [3]. The genome sequence of several sensu stricto species [4,5] revealed a level of nucleotide divergence comparable to that between humans and birds yet a level of structural variation comparable to that between humans and chimps [6]. Comparisons of genome structures have provided insight into mechanisms of genome evolution and speciation. For example, the presence of a limited number of genomic rearrangements that are not consistent with the phylogeny, provide strong evidence against the chromosomal speciation model [7].

Sequence comparisons between the sensu stricto species have allowed improved genome annotation [4,5]. Sequence conservation allowed the identification of additional small open reading frames and the refinement of translation start and stop positions. Lack of sequence conservation resulted in the elimination of spurious open reading frames. Combining experimental data for protein binding sites with sequence conservation allowed the identification of functional DNA sequences [8,9]. The power of these and other comparative genomic approaches [10] rely upon the number of species sequenced, the evolutionary divergence of the selected species and the quality of the assembled genome sequence.

Recently the yeast *Saccharomyces arboricolus* was isolated from the bark of the *Fagaceae* tree in China [11]. The *S. arboricolus* karyotype is consistent with the other sensu stricto species in terms of chromosome number and size. Sequence information (limited to a portion of the rDNA) unambiguously grouped this species within the sensu stricto complex. *S. arboricolus* can form viable hybrids with the other sensu stricto species but resulting gametes are not viable [12]. Together these data demonstrate that *S. arboricolus* is a novel sensu stricto species.

Here, we report high-quality sequence and assembly of the *S. arboricolus* genome (type strain H-6^T^; CBS 10644^TT^) by combining two deep sequencing platforms. We report chromosome size scaffolds, genome annotation and synteny analysis. Genome wide phylogenetic analysis places *S. arboricolus* between *S. bayanus* and *S. kudriavzevii* in the sensu stricto phylogenetic tree. Finally, we considered the phenotypic profile of *S. arboricolus* under multiple environmental conditions in the light of its gene content and phylogeny.

## Results

### Genome sequence and assembly

We generated a high quality genome assembly for *S. arboricolus* using a combination of high-throughput sequencing platforms and strategies (Table 1). First, we generated single-end reads using the Roche 454 pyrosequencing platform. This gave long reads that facilitated assembly. Second, we used Roche 454 paired-end reads, with ~8 kb insert size, to join contigs into chromosome size scaffolds (combined Roche 454 sequence coverage ~49X). We anticipated that the large insert size of the paired-end library would be sufficient to span any repeat elements (e.g. full length single Ty elements). Finally, we used 50 bp reads from SOLiD (Life Technologies) sequencing (~100X sequence coverage) to correct homopolymer errors present in the Roche 454 sequence. This combination strategy resulted in high quality sequence with chromosome-sized scaffolds.

**Table 1:** Deep sequencing metrics

**Library**	**Reads**	**Mapped reads**	**Mean mapped read length (bp)**
Roche 454 Fragment	734,353	726,488	360
Roche 454 8 kb Paired	1,711,390	1,520,755	200
Life Technologies SOLiD	31,316,590	21,753,029	50

*De novo* assembly of the Roche 454 (fragment and pair-end) reads was performed using the Newbler algorithm (see Methods). This resulted in 290 contigs (≥500 bp; N50 117,280 bp) that were joined using the Roche 454 paired-end reads to give 35 scaffolds. There are 17 scaffolds that are comprised of a single contig (2024 - 5644 bp) and one scaffold comprising of two contigs (9948 bp; Additional file 1: Table S1). The remaining 17 scaffolds account for >99% of the assembly and are between 72 and 1246 kb long. The total base count of the assembly, 11.6 Mb, is comparable to the physical genome size predicted from the karyotype [11] and is similar to the completed *S. cerevisiae* genome sequence (12.1 Mb) and genome sequence of other sensu stricto yeasts (11.6 - 11.9 Mb) [3,13].

**Figure 1:**
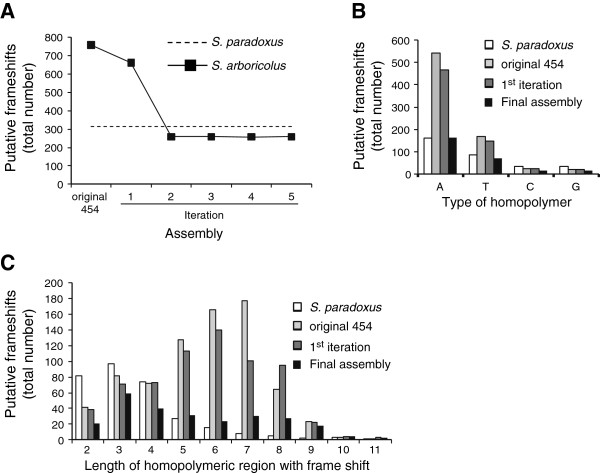
**Solving the homopolymer problem.** (**A**) Graph showing the number of frameshifts identified in each *S. arboricolus* assembly (filled symbols) compared to the *S. paradoxus* assembly (dashed trace). (**B**) Bar chart showing the number of frameshifts caused by homopolymers identified in each *S. arboricolus* assembly (filled bars) for each base compared to the *S. paradoxus* assembly (unfilled bars). (**C**) Bar chart showing the number of frameshifts caused by homopolymers of different lengths in each *S. arboricolus* assembly (filled bars) compared to the *S. paradoxus* assembly (unfilled bars).

Pyrosequencing suffers from an inherent difficulty in determining the number of incorporated nucleotides in homopolymer regions, due to the non-linear signal from the incorporation of >5 identical nucleotides [14]. Comparing our *S. arboricolus* genome sequence to *S. cerevisiae*, identified >700 open reading frames (ORFs) with putative frame-shifts (Figure 1A). These putative frame-shifts are predominantly in homopolymer runs and are therefore likely due to errors in the pyrosequencing (Figure 1). Indeed the *S. paradoxus* assembly [15], which was based on Sanger sequence reads (that do not suffer from homopolymer errors), shows less than half the number of frame-shifts than *S. arboricolus* (Figure 1A). We further analyzed the homopolymeric runs that cause frame-shifts and found that they tended to be longer and more A-biased than the corresponding frame-shifts in *S. paradoxus* (Figure 1B and C). To overcome this problem we used SOLiD sequencing that relies on a different chemistry and is not subject to the same error. We used the Roche 454 *de novo* assembly to map the SOLiD reads, identify errors and then correct the assembly. This mapping, errors calling and correction process was then repeated a further 4 times. In total we corrected 121 single base substitutions and 1682 indels. This resulted in a dramatic reduction in the number of putative frame-shifts to levels comparable to that seen with conventional Sanger sequencing (as represented by the *S. paradoxus* genome, Figure 1A). The corrected assembly also improved the distribution of frame-shifts such that the over-abundance of long homopolymeric runs and the A-bias were greatly reduced (Figure 1B and C).

The coverage of mapped SOLiD reads gives a measure of sequence copy number and can be used to reveal repeat regions that have collapsed during assembly. Overall we find a scarcity of high-coverage regions (Additional file 2: Figure S1), implying that there has been very little collapse of repeat regions during assembly. Short (<100 bp) regions of elevated copy number frequently correspond to highly repetitive tandem repeats and/or homopolymer tracks. Longer regions of elevated copy number are predominantly subtelomeric with the noteworthy exception of the rDNA repeats on chromosome XII.

The resulting *S. arboricolus* genome assembly comprises of whole chromosome scaffolds with only 186 gaps. These gaps have an average size of 1206 bp, the smallest two are just 1 bp, and the largest is 5846 bp. These regions consist of complex or repetitive sequences resulting in poor mapping of the SOLiD data. By comparison to recently improved assemblies of other sensu stricto yeasts [3], our *S. arboricolus* genome sequence has a higher proportion of the sequence (>99% compared with 96-98%) in a smaller number of scaffolds (35 compared with 147-226). Therefore, after the ‘gold standard’ *S. cerevisiae*, our *S. arboricolus* genome sequence represents the next most complete assembly.

### Genome structure and annotation

**Figure 2:**
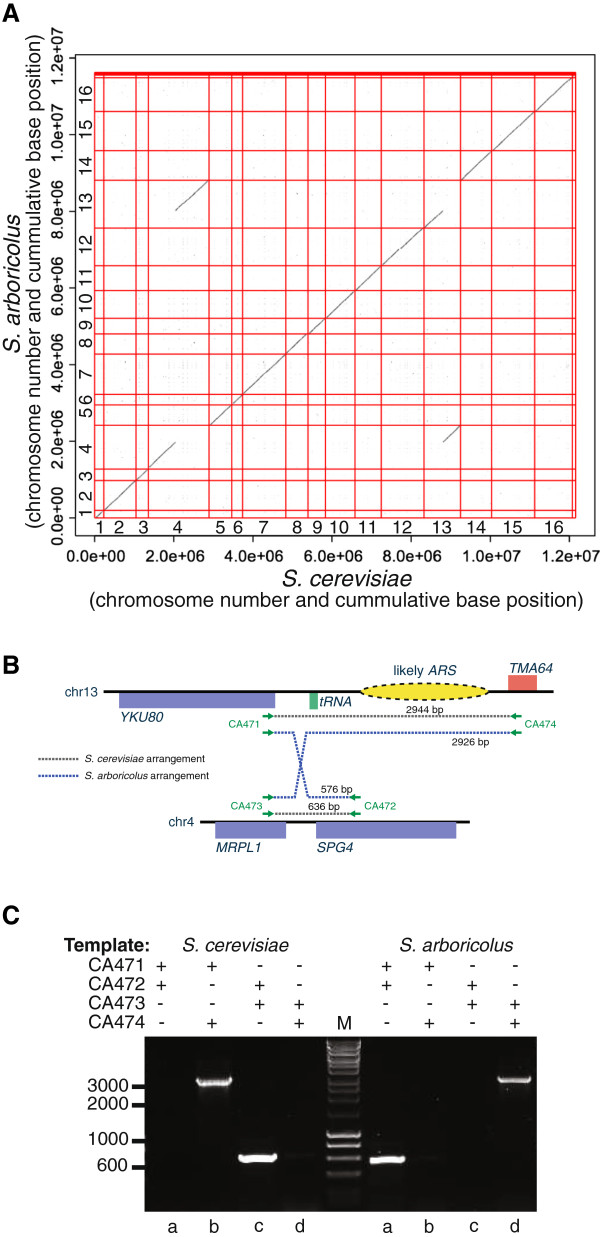
**Identification of a single reciprocal translocation.** (**A**) Dot plot representation of DNA sequence identity between the *S. cerevisiae* and *S. arboricolus* genomes. A single reciprocal translocation is apparent between chromosomes IV and XIII. (**B**) Cartoon representation of the location of the reciprocal translocation including the flanking features (red: Watson strand ORFs; blue: Crick strand ORFs; green: tRNA genes; yellow: autonomously replication sequence (ARS) [16,17]) and the primer locations (not to scale) used to confirm the translocation. (**C**) PCR-based confirmation of the reciprocal translocation. Various primer and template combinations (as indicated) were used to amplify products corresponding to either the *S. cerevisiae* or the *S. arboricolus* gene order. In each case the resulting PCR products support the reciprocal translocation identified by the genome assembly.

We compared our *S. arboricolus* genome assembly to the *S. cerevisiae* reference genome using LASTZ. We found that the 17 long scaffolds are each syntenic with a single *S. cerevisiae* chromosome or the mitochondrial genome with the exception of one predicted reciprocal translocation (Figure 2A) between the right arms of chromosome IV and XIII. The breakpoints are intergenic regions between ORFs *MRPL1* and *TMA64* on chromosome IV and *YKU80* and *SPG4* on chromosome XIII (Figure 2B). Interestingly, the breakpoint on chromosome XIII is adjacent to a tRNA gene, a feature previously reported to be associated with reciprocal translocations [7]. We used diagnostic PCR to experimentally confirm this reciprocal translocation (Figure 2C). The reciprocal translocation is unique to *S. arboricolus*, it is not present in *S. bayanus* or other sensu stricto assemblies [3] and therefore occurred after the *S. arboricolus* radiation.

The gene content of sensu stricto budding yeast species is thought to be similar [18,19], therefore we used comparative gene-annotation methods based on the well-annotated *S. cerevisiae* proteome to identify and annotate the ORFs in the *S. arboricolus* genome. Using exonerate [20], we aligned each *S. cerevisiae* protein to the *S. arboricolus* genome (see Methods). We assigned the top matching *S. arboricolus* ORF (based on the exonerate score) as a putative ortholog to each *S. cerevisiae* protein. We then compared the neighbouring genes of each *S. cerevisiae* gene with the neighbours of the putative orthologous *S. arboricolus* ORF to define a first set of 4798 orthologous gene pairs where the gene order has been conserved, which we refer to as “syntenic orthologs”. Because this method uses the best sequence match, missing assignments of syntenic orthologous ORFs may occur when the match with greatest sequence similarity is not the syntenic ortholog. To overcome this problem, we again used exonerate but emphasized the position of the predicted ORF, allowing the score to be slightly below the best scoring match (see Methods). An additional 519 *S. cerevisiae* genes had a high-scoring, but not top match with the expected syntenic gene pair. We considered these to be syntenic orthologs as sequence similarity together with gene order conservation is thought to be a more reliable indicator of orthology than sequence similarity alone [21]. To identify genes that may be found in *S. arboricolus*, but not in *S. cerevisiae*, we used Genemark [22], which is a *de novo* gene prediction method, and does not rely on sequence similarity (see Methods) and identified 106 genes that were not predicted using exonerate. These Genemark predictions contain novel genes and ORFs that were missed by exonerate as only the best hit from exonerate was considered in our gene prediction.

**Figure 3:**
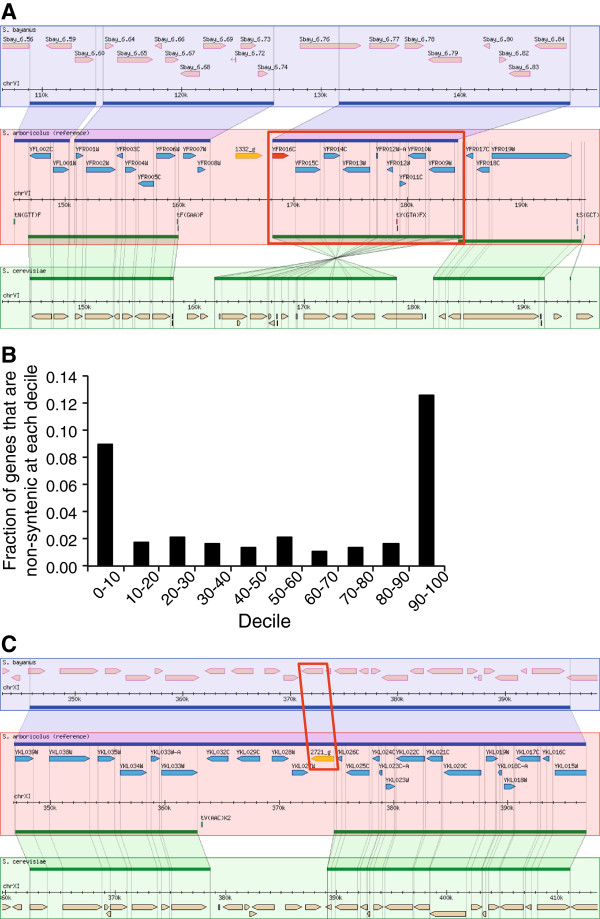
**Annotation of the *S. arboricolus *genome.** (**A**) A Gbrowse_syn visualization of the inversion on chromosome VI within *S. cerevisiae* with respect to *S. arboricolus* and *S. bayanus*. (**B**) Novel and non-syntenic genes are predominantly telomeric. Each chromosome was divided in deciles and the fraction of genes predicted at each decile that we define as novel and non-syntenic is shown. (**C**) Gene complement differences from *S. cerevisiae* can be explained by *S. cerevisiae* specific gene loss. A Gbrowse_syn visualization, showing a gene (labeled as 2721_g) present in both *S. bayanus* and *S. arboricolus*, but not in *S. cerevisiae*. In the Gbrowse_syn visualizations *S. bayanus* genes are shown in pink, *S. arboricolus* genes in blue and *S. cerevisiae* genes in orange; *S. bayanus* - *S. arboricolus* syntenic regions are marked by blue horizontal lines; *S. arboricolus* - *S. cerevisiae* syntenic regions are marked by green horizontal lines.

**Figure 4:**
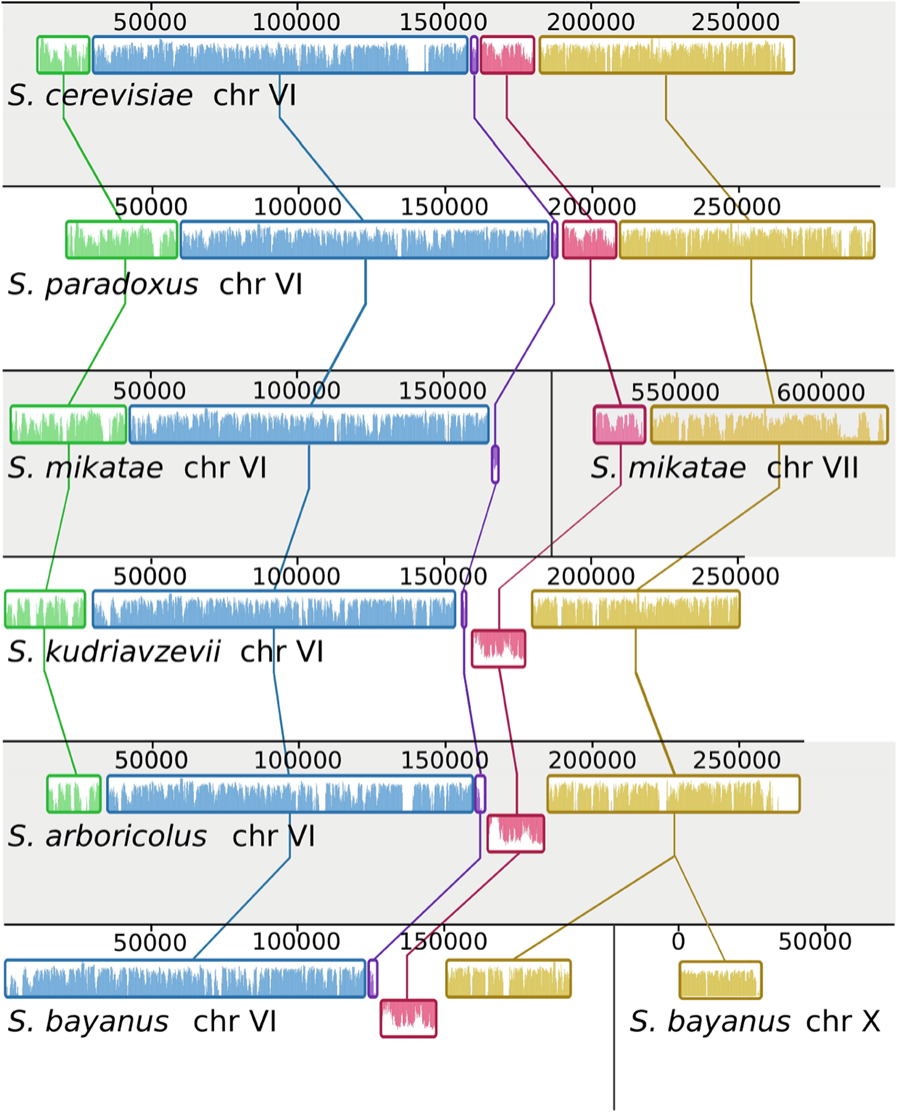
**Structure of *Saccharomyces *sensu stricto chromosome VI.** Chromosomal blocks of high sequence similarity are given the same colour and are connected by vertical lines. The average conservation level of the sequence is displayed within each block. Blocks placed below the horizontal center of each chromosome are showing sequence similarity in the inverse direction. In *S. mikatae* and *S. bayanus*, parts of chromosome VI have been translocated to chromosome VII and chromosome X, respectively [7].

We explored the possibility that our annotation of the *S. arboricolus* genome contained novel genes. As was observed with the *S. bayanus* genome [19], the vast majority (96%) of the genes in *S. arboricolus* have conserved gene order with *S. cerevisiae*. The remaining “non-syntenic” genes include 104 that have similarity to another *S. cerevisiae* gene but are not syntenic (by our definition) and the 106 the genes predicted *de novo* (within the 16 assembled chromosomes). Analysis of the non-syntenic genes allowed the detection of at least two small local rearrangements relative to *S. cerevisiae* due to inversion of a large portion of DNA. The first one occurs on chromosome VI between ORFs *FAR7* and *YFR017C* (Figure 3A) and the second one on chromosome XIV between *YNL034W* and *COG6* (data not shown). To determine whether these were specific to the *S. arboricolus* genome, we compared these regions to the other sensu stricto genomes, and found that the *S. cerevisiae* gene order is likely to be the derived state, as *S. bayanus* and *S. kudriavzevii* shows the same gene order as *S. arboricolus* (Figure 4). Other synteny breaks occur predominantly in the subtelomeric regions: there is a significant enrichment of non-syntenic and novel genes predicted in the first and last 10% of the chromosomes (P-value = 5×10^-37^, Figure 3B) [23]. We also considered the genes predicted in *S. arboricolus* that were not syntenic with *S. cerevisiae*. Of these 210 genes, 44 had no BLAST hits within the *S. cerevisiae* genome (e-value cutoff 1e-10). Interestingly, 3 of these 44 genes are likely to be *S. cerevisiae* specific gene losses, rather than new genes arising in *S. arboricolus*, as they are found in *S. bayanus* (Figure 3C). Two of the non-telomeric *S. cerevisiae* gene losses are *SIR1* genes as previously reported [24]. Of the remaining 41 genes, 20 have no blast hit within Uniref90 (e-value cutoff 1e-10) and we considered the possibility that these were truly novel genes. After manual inspection based on presence of stop codons within the predicted peptide, protein sequence lengths, Pfam analysis andadditional blast searches, we concluded that 4 of these genes are likely to represent novel genes in *S. arboricolus* (Additional file 3).

We also searched for tRNA coding sequences within the 16 chromosomes using tRNAscan-SE [25] and annotated whether they were syntenic using a similar strategy to that described above (see Methods). In total, 257 tRNAs were found, 252 of which are syntenic with *S. cerevisiae* tRNAs. Next we used BLAST to search for the presence of repetitive elements in the genome such as subtelomeric genes and Ty elements. We detected the most distal subtelomeric element, Y’, in the genome sequence. This element is therefore present in all the sensu stricto species except *S. bayanus*[26,27]. We also detected Ty2 element sequences using as a query the region that does not share similarity with the Ty1 element (1.7-kb *ClaI* Ty2- specific sequences [26]).

### Phylogenetic analysis

**Figure 5:**
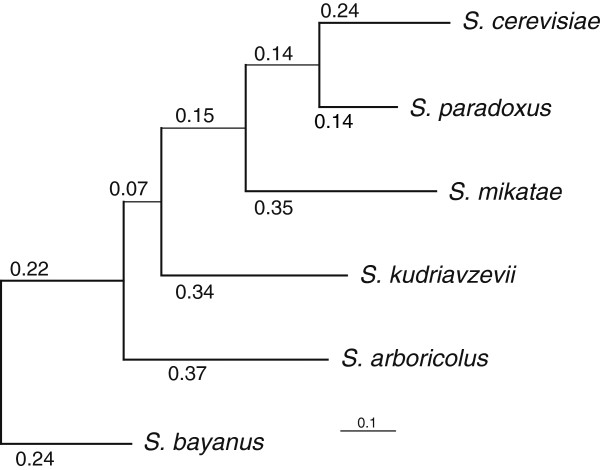
**Phylogenetic analyses of the *Saccharomyces *sensu stricto group.** A phylogenetic tree of the sensu stricto *Saccharomyces* species places *S. arboricolus* between *S. bayanus* and *S. kudriavzevii*. Tree topology was obtained using random concatenations of protein sequences with *S. castellii* as the outgroup. Branch lengths represent the maximal likelihood estimate of the evolutionary distance in DNA substitutions per codon using all genes that we identified as ‘syntenic orthologs’.

We tested five possible placements of *S. arboricolus* within the sensu stricto complex (Additional file 4: Figure S2), by sampling 100 sets of 50 random proteins for which we have data for all 6 sensu stricto species, as well as *S. castellii* as outgroup. These protein sequences were concatenated, and we computed the likelihood of the five phylogenetic trees using PAML. All 100 trees supported the grouping of *S. arboricolus* as diverging after the common ancestor with *S. bayanus* and before *S. kudriavzevii*, and all but 1 of these trees obtained bootstrap scores >0.9. Originally *S. arboricolus* was placed, based on a limited amount of ribosomal DNA sequence, between *S. mikatae* and *S. kudriavzevii*[11], however our genome-scale phylogenetic analysis has much greater power and unambiguously supports the new tree structure (Figure 5).

We next set out to estimate the evolutionary distances between the species in the sensu stricto clade. To do so, we used the phylogeny as determined above, and computed codon-based maximum likelihood estimates (see Methods) of evolutionary distance based on alignments of 3899 genes for which we had 1 to 1 orthologs in all of the sensu stricto species, and that were syntenic between *S. arbicolus* and *S. cerevisiae*. We computed the median branch lengths (in substitutions *per* codon) for these, and they are shown in Figure 5.

### *S. arboricolus* web and strain resources

To make the *S. arboricolus genome* sequence available and to facilitate analysis, we have made available a number of web resources (http://www.moseslab.csb.utoronto.ca/sarb). These include genome sequence, annotation and datasets for genes and proteins. The gene and protein sets are annotated based on the *S. cerevisiae* ortholog systematic name. Novel putative ORFs identified by Genemark are also reported. A BLAST server and a genome browser (Gbrowse [28] and GBrowse_syn [29]) are available. The *S. arboricolus* genome browser offers the opportunity to view and compare the genome structure of *S. cerevisiae*, *S. arboricolus* and *S. bayanus*. Using *S. arboricolus* as the central reference species, gene order conservation or chromosomal rearrangements between the three species can easily be observed. Genes in *S. arboricolus* are coloured differently based on their annotation (e.g. syntenic orthologs, Genemark predictions, etc.). Finally, to facilitate experimental analysis of *S. arboricolus* the *HO* gene was disrupted (in the type strain H-6) and stable haploids were generated.

### *S. arboricolus* phenotypic landscape

**Figure 6:**
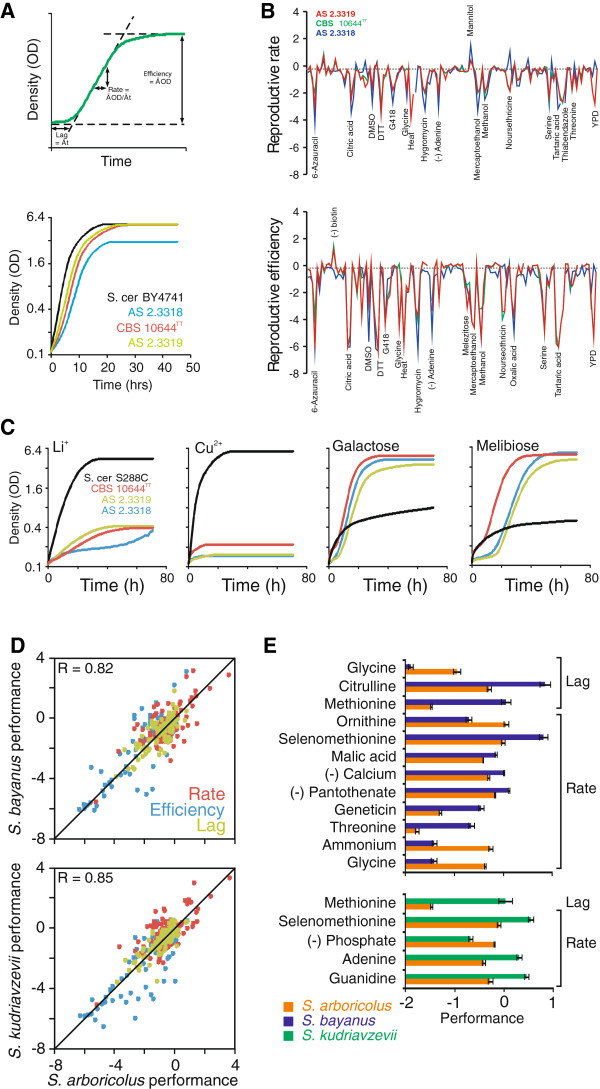
**Phenomics analyses of *S. arboricolus. *** (**A**) Reproductive lag, rate (population doubling time) and efficiency (change in population density) of *S. arboricolus* CBS 10644^TT^, AS 2.3317 and AS 2.3319 were extracted from high density growth curves in no stress conditions. The performance of the *S. cerevisiae* strain BY4741 is shown as reference. (**B**) Relative reproductive performance of *S. arboricolus* strains CBS 10644^TT^, AS 2.3317 and AS 2.3319 in a wide array of environments. The performance of each strain (n=2) was normalized to the *S. cerevisiae* reference strain BY4741 (n=20), or its auxotrophic mother S288C, providing a relative measure (log_2_ [BY4741/isolate]). Broken line shows average performance in basal (no stress) conditions. Strong phenotype deviations from *S. cerevisiae* are labeled with the respective condition. (**C**) Mitotic reproduction of *S. arboricolus* strains during conditions of elevated concentrations of Li^+^ (0.3 M) and Cu^2+^ (1 mM) or utilizing galactose and melibiose as sole carbon sources. *S. cerevisiae* S288C is included as a reference. (**D**) Average reproductive lag, rate and efficiency of *S. arboricolus* (CBS 10644^TT^, AS 2.3317 and AS 2.3319) plotted against the corresponding averages for *S. bayanus* (CBS1001, GL274 and GL388) and *S. kudriavzevii* (GL22, GL23, GL391 and GL392) [30]. Red = lag, green = rate, blue = efficiency. Grey diagonals indicate 1:1 correlations, numbers indicate Pearson correlation coefficients. (**E**) Phenotypes distinguishing *S. arboricolus* from *S. bayanus* and *S. kudriavzevii* respectively. Significant phenotype differences were defined at α<0.2 (Students *t*-test, Bonferroni correction). Error bars = Standard Error of the Mean.

Taking the phylogeny and gene content of *S. arboricolus* (described above) into account, we revisited recently generated data on its phenotypic diversity. Although included for completeness in our publication on the phenotypic landscape of *Saccharomyces* sensu stricto species [30], the *S. arboricolus* phenotypes were not specifically analyzed or commented on. The sequenced strain (CBS 10644^TT^) and two additional, genetically distinct lineages isolated from similar habitats in Southern China were subjected to high resolution phenotyping of proliferative capability across >120 environments selected to represent variations in common yeast habitats, such as carbon and nitrogen source variations, tolerance to metabolites and toxins produced by plants and bacteria, and variations in vitamins and mineral availability (Additional file 5: Table S2). The fitness components lag, rate (population doubling time) and efficiency of reproduction (population density change) were extracted from high density growth curves and normalized to those of the *S. cerevisiae* reference strain, providing >360 precise measures of organism-environment interactions (Figure 6A). In the absence of stress, *S. arboricolus* proliferated slightly slower than *S. cerevisiae* and one strain (CBS 10644^TT^) also showed reduced efficiency (Figure 6A). However, these *S. arboricolus* growth aberrations in conditions with no stress were marginal compared to the dramatic proliferation deviations observed in a vast range of stress-inducing niche environments (Figure 6B). Remarkably, almost all of these aberrations constituted grave defects, many corresponding to more than 10-fold reductions in mitotic performance. Thus, *S. arboricolus* showed drastically reduced tolerance to fruit organic acids such as citric, tartaric and oxalic acid and to high temperatures and very poor utilization of adenine, serine and threonine as nitrogen sources. Notably, *S. arboricolus* failed to proliferate during conditions of elevated Li^+^ and Cu^2+^, traits likely explained by absence of the amplifications of the lithium exporter (*ENA1*) and the copper metallothionein (*CUP1*) that determine these traits in *S. cerevisiae*[30] (Figure 6C). The many niche specific proliferation deficiencies of *S. arboricolus* may explain its limited geographical and ecological distribution compared to *S. cerevisiae*.

Among the rare examples of superior *S. arboricolus* performance were better utilization of the sugar alcohol mannitol, one of the most abundant energy storage molecules in nature [31] and tolerance to biotin depletion, rarely observed in *S. cerevisiae* due to ancestral loss of the biotin synthesis genes *BIO1* and *BIO6*[32]. Both *BIO1* and *BIO6* are present as conserved cistrons in *S. arboricolus* strain CBS 10644^TT^ (Figure 6B), explaining the biotin auxotrophy. *S. arboricolus* also featured consistently good utilization of the monosaccharide galactose (Figure 6C), a highly variable trait in both *S. cerevisiae*[30] and *S. kudriavzevii*[33] due to frequent loss-of-function mutations emerging in different lineages of these species that impair growth on galactose (e.g. the reference strain S288C). The coding sequences of the GAL pathway genes are also fully conserved in *S. arboricolus* strain CBS 10644^TT^. Interestingly, *S. arboricolus* strain CBS 10644^TT^ has also retained an intact melibiase encoding *MEL1*, which is lost in most *S. cerevisiae* lineages [30]; all three *S. arboricolus* isolates also utilized the disaccharide melibiose, a less common plant energy storage compound, with a vastly superior rate and efficiency.

Overall, the three *S. arboricolus* isolates showed virtually identical trait profiles (Pearson correlation, r=0.73-0.91). Moreover, using data from Warringer et al. [30], we found *S. arboricolus* traits to closely mimic those of its relatives, *S. bayanus* and *S. kudriavzevii* (Figure 6D). These remarkable trait similarities, encompassing mitotic performance in a wide variety of environments, imply that these three species, despite billions of generations of separation, are adapted to rather similar ecological conditions. The few cases of *S. arboricolus* deviations from *S. bayanus* and *S. kudriavzevii* primarily included nitrogen utilization traits, such as superior *S. arboricolus* utilization of ammonium, glycine and ornithine, but inferior utilization of methionine, serine and citrulline (Figure 6E). Presumably, this reflects differences in nitrogen storage compounds among plant species that dominate the main habitats of these species and hint at ecological factors that may have driven speciation of the ancestral lineages. Further indications as to the nature of these factors may also be found in the reduced tolerance of *S. arboricolus* to malic acid, concentrated in e.g. apples, and to the toxin geneticin, produced by bacteria of the *Micromonospora* genus (Figure 6E).

## Discussion

Our approach of using multiple high-throughput sequencing strategies resulted in high quality genome sequence that continuously covers the large majority of the *S. arboricolus* genome. The *de novo* assembly revealed that *S. arboricolus* is largely syntenic to *S. cerevisiae*, similar to the other sensu stricto species. We mapped and validated a single reciprocal translocation that occurred in the *S. arboricolus* lineage and identified a few additional small-scale rearrangements. Our assembly extends into the subtelomeric regions of most chromosome ends. However, these repetitive sequences pose a major challenge to genome assemblies and the subtelomeric structure presented here will benefit from further experimental validation.

Sequence analysis unambiguously revealed the position of *S. arboricolus* in the phylogenetic tree (Figure 5; correcting the previously reported position based on a limited amount of ribosomal sequence [11]). Phylogeny of individual genes revealed a limited number of conflicting tree topology as has been previously reported for other sensu stricto species [34]. We did not observe large segments of the genome (for example, equivalent in size to the average gene) with high similarity with other species as signature of introgression as previously reported in other species and strains [35-37].

We have looked for the presence and absence of middle repetitive elements such as Ty and subtelomeric genes. For some of these elements the presence and absence is consistent with the phylogeny [26]. We detected the subtelomeric element Y’ in *S. arboricolus*, indicating that this element entered in the sensu stricto ancestor after the divergence of *S. bayanus*. Much more puzzling is the phylogeny of Ty2, present in *S. cerevisiae*, *S. mikatae* and *S. arboricolus* but absent in *S. paradoxus, S. kudriavzevii* and *S. bayanus*. Both multiple loss and acquisition can explain the scattered phylogeny but are unlikely events. One possibility is a recent exchange of Ty2 among these species (horizontal transfer) as supported by high sequence similarity. A possible mechanism is the ability of these species to fuse their cytoplasms, without progressing to karyogamy, and allowing the exchange of Ty particles that can self-propagate in the genome.

Phenotype analysis demonstrated a remarkable similarity in trait profiles between *S. arboricolus* and both *S. bayanus* and *S. kudriavzevii* (Figure 6D). These similarities suggest that these three species have adapted to similar environmental niches. The limited number of phenotypic differences between these species may reflect the specific nutrients available within each species habitat. Phenotypic comparisons between *S. cerevisiae* and *S. arboricolus* frequently reflect differences in gene content, including the sensitivity of *S. arboricolus* to elevated Li^+^ and Cu^2+^ and the ability of *S. arboricolus* to utilize melibiose.

So far, the Chinese isolates of *S. arboricolus* are the only ones available. Future surveys will reveal whether this species is limited to this region or whether other geographic populations exist. It is interesting to note that two of the *Saccharomyces* species have only been isolated in Asia (*S. mikatae* in Japan and *S. arboricolus* in China) despite extensive surveys in similar environments in other continents [38-40].

## Conclusions

The *Saccharomyces* sensu stricto complex offers a powerful range of sequence divergences that have allowed the mapping of functional elements [4,5], improved genome annotation and comparisons of genome organization [7,19,41]. Genome sequencing has revealed levels of divergence ranging from 0.1 - 0.6% among *S. cerevisiae* strains, 1.5 - 4.5% between geographic subpopulations of *S. paradoxus*[15], 6% between *S. bayanus var. uvarum* and *S. eubayanus*[42], and 15% - 30% between *S. cerevisiae* and the other sensu stricto species [4,5]. The *S. arboricolus* genome sequence should enhance the power of comparative genomics by increasing the total sequence divergence and improve the quality of alignments by adding a new branch between *S. bayanus* and *S. kudriavzevii*, the more divergent species.

## Methods

### Genomic DNA and library preparation

We extracted DNA from the type strain of *S. arboricolus* H-6^T^ (CBS 10644^TT^) isolated in China from the bark of *Quercus fabri*[11]. For the Roche 454 library construction and sequencing, 5 μg of high molecular weight genomic DNA was used to make standard shotgun DNA library as described in the Roche GS FLX Titanium General Library Preparation Method Manual with the exception of DNA fragmentation, which was done with Covaris S2 sonicator (fragmentation parameters: Duty cycle - 5%, Intensity - 1, cycles/burst - 200, time - 85 s, bath temperature - 5°C). 15 μg of high molecular weight genomic DNA was used to make the 8 Kb paired end library as stated in the Roche GS FLX Titanium 8 Kb Span Paired end library preparation method manual. Exceptions include: DNA extraction was done using QIAquick Gel extraction kit (Qiagen, Cat no. 28760) instead of Electroelution as stated in the manual and fragmentation of circularised DNA was done using Covaris S2 sonicator (Duty cycle −5%, Intensity - 3, cycles/burst - 200, time - 120 s, bath temperature - 5°C). Sequencing of standard shotgun fragment library was carried out on ¾ of a PTP and the 8 Kb paired end library was sequenced on a full PTP using Roche 454 Titanium sequencing chemistry. For SOLiD library construction and sequencing, 500 ng of high molecular weight genomic DNA was used to make a barcoded DNA fragment library as stated in the SOLiD 4 library preparation guide. Enzymes and reagents were used from NEBNext DNA sample prep Master mix set 3 (NEB, Cat no. E6060L). The barcoded DNA fragment library was quantified using Kapa Library Quantification kit (Kapa Biosystems, Cat no. KK4823). 200-300 bp library size selection was carried out using 2% SizeSelect E-Gel (Life Technologies, Cat no. G6610-02). SOLiD EZ Bead System was used according to manufacturer’s guide to prepare ePCR and templated bead enrichment. Sequencing was performed on a SOLiD 4 analyser according to the manufacturer’s instructions to generate 50 bp reads in colour space.

### Genome assembly

We assembled the genome of *S. arboricolus* using the Newbler algorithm (v2.3, Roche) for *de novo* assembly of reads generated by the 454 pryosequencing platform. Combinations of read datasets, reads added in assembly iterations, and assembler parameters were explored before selecting the optimal combination according to assembly metrics (number of scaffold sequences and contigs, the average and longest contig length and N50 value). All reads were trimmed against a dataset of adapter and vector sequences in the initial step of the assembly process.

The selected assembly parameters used an expected coverage value of 40X with all other settings remaining at default values. Two assembler iterations were employed; the first iteration included all 734,353 single fragment reads and one set of 583,674 paired-end reads. The second iteration incorporated an additional set of 518,434 paired-end reads. A third set of paired-end reads was excluded from the assembly due to decreased performance with their inclusion.

The resulting genome assembly comprised of 32 scaffold sequences with a total length of 11,465,281 bp. The scaffolds were comprised of 266 contigs (≥500 bp) with an average length of 43,102 bp (538,482 bp max.) and an N50 value of 136,945 bp. The mapped read coverage of the assembly was 49X.

### Pyrosequencing error correction

In order to resolve small errors in the assembly arising from pyrosequencing artifacts, such as homopolymer sequence regions [43,44], we acquired deep sequence coverage (~100X) from short reads. We generated a total of 31,316,59 short (50 bp) reads from a SOLiD 4 single fragment library. Subsequent gapped read alignment and variant calling was achieved using Bioscope 1.3.1 (Life Technologies).

An iterative correction process was devised in which errors in the assembled sequence were identified from the SOLiD read alignment data as either a single nucleotide polymorphisms (SNP) for single base errors, or as small InDels (insertion/deletion) for homopolymer pyrosequencing errors. Each iteration of the assembly correction process involved the initial mapping of SOLiD reads against the 454 assembly, followed by SNP calling. Selected putative SNPs were then integrated into the assembly sequence and SOLiD reads were remapped to allow InDels to be called and integrated. This process was repeated until no additional variants were detected. In subsequent iterations additional reads were mapped allowing the identification and correction of a small number of further errors. Both SNPs and InDels were calculated from alignment data using Bioscope 'high stringency' variant parameter settings. Additionally, integrated variants were required to represent a minimum of 60% of the alignment data.

### Gene annotation and orthology assignments

*S. cerevisiae* was used as the reference proteome for the program exonerate, which uses comparative approaches for gene finding based on protein sequence similarity. An initial pass with the protein2dna model and a refine boundary of 2000 was used to find the best orthologous candidate of each *S. cerevisiae* gene. For intronic genes, the max intron size was limited to 1500 bp and the model used was protein2genome.

To annotate genes within the *S. arboricolus* genome, we first identified gene orthologs with conserved synteny. To do so, we analysed the top hit by exonerate for each *S. cerevisiae* gene. When three neighbouring genes within *S. cerevisiae* all identified three neighbouring genes within *S. arboricolus*, we assigned the *S. arboricolus* gene in the middle (flanked by its two neighbours) as a syntenic ortholog. This initial step discovered most of the syntenic genes within *S. arboricolus*. Other genes within *S. cerevisiae* that had not been assigned an ortholog were further analysed with the hypothesis that these may have exonerate hits within the expected positions but were not the most similar sequence within *S. arboricolus*. We looked at the top 10 exonerate hits of the remaining *S. cerevisiae* genes for matches in *S. arboricolus* between the initially assigned syntenic ortholog. When only one hit was found between these syntenic orthologs, we used this hit as a newly discovered syntenic ortholog. This process was repeated until no more syntenic orthologs could be found. Finally, we assigned the top exonerate hit of few remaining *S. cerevisiae* genes that were still not assigned a syntenic ortholog as the non-syntenic ortholog provided that they did not overlap with another gene prediction.

*De novo* gene prediction on the *S. arboricolus* genome was performed using GeneMark-ES, version 2 [45]. The total number of the predicted genes was 5005 within the 16 assembled chromosomes (5038 in total). Of these, 95 genes had non-overlapping coordinates with the genes predicted by Exonerate within the 16 assembled chromosomes (106 in total when including the 19 scaffolds that did not assemble into the chromosomes).

A significant issue when using a comparative-based method, such as exonerate, for gene prediction is that gene boundaries are often incorrectly predicted if there is a lack of homology at these ends. Initially, a large number of predicted genes did not contain a start or stop codon (637 genes and 1121 genes respectively). We have attempted to rectify these starts and ends by extending or truncating the predicted CDS. First, CDSs were extended if a stop codon could be found within 9 codons from the end of our gene prediction. This corrected 857 cases of missing stop codons and further extension only slightly improved the annotation. Second, for start codons, the methionine can be on either side of the predicted gene start. We therefore extended the predicted gene until a methionine was found, but only when a methionine could be found within 9 codons and without any intervening stop codons. In the cases where a stop codon occurred before a suitable methionine was identified, we truncated the CDS to a downstream methionine if it occurred within 9 codons. This corrected 348 cases of missing start codons. Finally, intron-containing genes were left untouched as missing starts and ends for these genes could be due to a missing exon. We note that for intron-containing genes, we specifically use the protein2genome model in exonerate that explicitly attempts to predict all exons found in *S. cerevisiae* genes. This assumes that the presence of introns is conserved between *S. cerevisiae* and *S. arboricolus*.

We aligned the protein sequence orthologs for the sensu stricto using MAFFT [46] with default settings, either with or without *S. castellii* orthologs as an outgroup. For the coding sequence analysis we inserted the gaps back into the DNA sequences. Phylogenetic analysis was performed using PAML [47], either with the codon model for the DNA sequence analysis or with empirical model for the amino acid analysis. Because we are only concerned with the placement of *S. arboricolus* within the established sensu stricto yeast phylogeny, we compared the likelihood of several putative tree topologies that differ only in the position of *S. arboricolus* (Figure S2).

To annotate tRNA coding sequences, we predicted tRNAs using tRNAscan-SE [25] with default settings on the 16 assembled chromosomes. To determine whether or not these predicted tRNAs are syntenic with respect to *S. cerevisiae* we used an analogous strategy to that described above for gene annotations. tRNA coding sequences were annotated as syntenic orthologs if they were flanked by genes within *S. arboricolus* that were assigned as syntenic orthologs and if a tRNA was also found in *S. cerevisiae* between those genes. In all but one cases, the syntenic tRNAs code for the same amino acids.

### Chromosomal structure plots

Chromosome structure plots for the *Saccharomyces* sensu stricto species were constructed using Mauve [48]. Assembled chromosomes for *S. paradoxus* (strain CBS432) were obtained from [15] and for *S. mikatae* (IFO 1815 ^T^), *S. kudriavzevii* (IFO 1802 ^T^) and *S. bayanus var. uvaru*m (strain CBS 7001) from [3]. As these chromosome assemblies have been constructed partly by using the *S. cerevisiae* genome to orient and order scaffolds, alignments were also made to the unordered scaffolds using MUMmer [49] to confirm the relative orientation of chromosomal segments inverted between species.

### Mapping of the phenotype landscape of *S. arboricolus*

The bulk of the phenotypic data was taken from our recent publication [30] on sensu stricto phenotypes where it was included for completeness but where *S. arboricolus* phenotypes were not specifically analyzed or considered. The data displayed as growth curves in this study correspond to novel confirmatory runs performed to ensure the reliability of specific statements. Three diploid isolates of *Saccharomyces arboricolus* were collected as described previously [11] and long time stored in 20% glycerol at -80C. Isolates were subjected to high throughput phenotyping by micro-cultivation (n=2) in an array of environments (Additional file 5: Table S2) essentially as previously described [50]. For pre-cultivations, strains were inoculated in 350 μL of SD medium (0.14% yeast nitrogen base, 0.5% ammonium sulfate and 1% succinic acid; 2% (w/v) glucose; 0.077% Complete Supplement Mixture (CSM, ForMedium), pH set to 5.8 with NaOH or KOH) and incubated for 48 h at 30°C. For experiments where the removal of a specific media component was studied, the pre-culture was performed in absence of this component in order to completely deplete the component in question. For experiments where alternative nitrogen sources were used, two consecutive pre-cultures were performed, the first containing low amounts of ammonium sulphate (0.05%), the second replacing ammonium with the indicated nitrogen source in amounts corresponding to equivalent moles of N. For all experimental runs, strains were inoculated to an OD of 0.03 - 0.1 in 350 μL of SD medium and cultivated for 72 h in a Bioscreen analyser C (Growth curves Oy, Finland). Optical density was measured using a wide band (450-580 nm) filter. Incubation was at 30.0°C (±0.1°C) with ten minutes preheating time. Plates were subjected to shaking at highest shaking intensity with 60s of shaking every other minute. OD measurements were taken every 20 min. Strains were run in duplicates on separate plates with ten replicates of the universal *S. cerevisiae* reference strain BY4741 or its prototrophic mother S288C, in randomised (once) positions on each plate as a reference. The reproductive rate (population doubling time), lag (population adaptation time) and efficiency (population total change in density) were extracted from high density growth curves and put in relation to the corresponding fitness variables of the reference strain BY4741, or in conditions directly involving alterations of nitrogen content, its prototrophic mother S288C, as described previously [30]. The derived Log_2_ ratios (Log_2_ (BY4741/isolate) or, in case of efficiency, Log_2_ (isolate/BY4741) were used for subsequent analysis.

### Accession numbers

Raw sequencing reads are available from the European Nucleotide Archive (EBI ENA) for the SOLiD reads [EMBL: ERP001702], Roche 454 single fragment reads [EMBL: ERP001703] and Roche 454 paired-end reads [EMBL: ERP001704]. The assembled genome is available from NCBI as *Saccharomyces arboricola* [GenBank: ALIE00000000].

## Competing interests

The authors declare that they have no competing interest.

## Authors' contributions

JW carried out the phenotyping experiments. SM made the sequencing libraries. CAM, FD-C and FAC performed the remaining experiments. MB generated the genome assembly. ANNB, AB, CCS, NM and SK performed computational analysis. ANNB generated the web tools. GL, JW, AMM, EJL and CAN designed and supervised the experiments and computational analysis. GL and CAN wrote the manuscript. All authors read and approved the final manuscript.

## Supplementary Material

Additional file 1: Table S1 Sequence homology of the small scaffolds. Each of the 18 small scaffolds (<10 kb) was compared to the *S. cerevisiae* genome using BLAST. For each small scaffold the name, size and sequence homology are listed. Click here for file

Additional file 2: Figure S1 SOLiD coverage across the 16 chromosomes of *S. arboricolus* to detect regions of elevated copy number. There is one plot for each chromosome, with the x-axis representing the chromosomal coordinate and the y-axis (on a log2 scale) representing sequence coverage as a measure of copy number (normalized by the genome-wide average). SOLiD reads were mapped to the *S. arboricolus* assembly using BFAST [ [51]]. Reads with multiple equally good top scoring mapping locations were assigned randomly to one of these. The depth of coverage of reads mapping to the assembly was calculated in windows of size 1 kb along the chromosomes. Each step on the vertical axis corresponds to one unit on the logarithmic scale. Vertical bars in orange mark the locations of gaps in the assembly and bars in blue mark the locations of tandem repeat tracts longer than 30 bp, as predicted by Tandem Repeats Finder [ [52]]. Click here for file

Additional file 3: File S1 Fasta file containing the protein sequences of novel genes in *S. arboricolus*. Click here for file

Additional file 4: Figure S2 Five possible placements of *S. arboricolus* within the sensu stricto complex. We find that the third placement is supported by the data (main manuscript Figure 5). *S. cer*: *S. cerevisiae*; *S. par*: *S. paradoxus*; *S. mik*: *S. mikatae*; *S. kud*: *S. kudriavzevii*; *S. arb*: *S. arboricolus*; and *S. bay*: *S. bayanus*. Click here for file

Additional file 5: Table S2 Environments used in the phenotyping screen. Classification”carbon utilization” indicates that 2% glucose was substituted with the indicated carbon source, classification “nitrogen utilization” indicates that 0.5% ammonium sulfate was substituted with the indicated nitrogen sources at nitrogen limiting concentrations. In all nitrogen utilization experiments, two consecutive pre-cultures were performed to deplete internal nitrogen storages: the first with nitrogen limiting amounts of ammonium, the second with nitrogen limiting amounts of the indicated nitrogen source. # = pre-cultures were performed in medium similar to the experimental medium to deplete internal storages of the molecule. Click here for file
