# Supplementary material for: High quality de novo sequencing and assembly of the Saccharomyces arboricolus genome
Source: BMC Genomics. 2013 Jan 31;14:69. doi: 10.1186/1471-2164-14-69 (PMC3599269; doi:10.1186/1471-2164-14-69)
Supplement: Additional file 1: Table S1 — Sequence homology of the small scaffolds. Each of the 18 small scaffolds (<10 kb) was compared to the S. cerevisiae genome using BLAST. For each small scaffold the name, size and sequence homology are listed. [file 1471-2164-14-69-S1.docx]

**Supplementary Table S1. Sequence homology of the small scaffolds.** Each of the 18 small scaffolds (<10 kb) were compared to the *S. cerevisiae* genome using BLAST.

| Scaffold | Length (bp) | Sequence homology |
| --- | --- | --- |
| scaffold2_01 | 9948 | Homology to *S. cerevisiae HXT17**. |
| scaffold2_02 | 5644 | Homology to *S. cerevisiae YLR455W*, *YLR456W*, *NBP1* & *CDC91**. |
| scaffold2_03 | 4546 | Homology to part of *S. cerevisiae* Y' element*. |
| scaffold2_04 | 3981 | Homology to *S. cerevisiae PHO85* & *YPL030W*. |
| scaffold2_05 | 3783 | Homology to *S. cerevisiae SEC4* & part of *BLM3*. |
| scaffold2_06 | 3738 | Homology to *S. cerevisiae RAS2* & *PHO23*. |
| scaffold2_07 | 3152 | Homology to part of *S. cerevisiae PDR5*. |
| scaffold2_09 | 2605 | Homology to *S. cerevisiae* LTR from Ty2 / Ty1 elements. |
| scaffold2_10 | 2595 | Homology to part of *S. cerevisiae FSP2* (and other homologues)*. |
| scaffold2_11 | 2554 | Homology to part of *S. cerevisiae YNR034W-A* & *ARC35*. |
| scaffold2_12 | 2436 | Homology to *S. cerevisiae EFT1* & *EFT2*. |
| scaffold2_13 | 2316 | Homology to *S. cerevisiae YKL151C* & part of *MCR1*. |
| scaffold2_14 | 2140 | Homology to *S. cerevisiae YNL035C*, *tRNA* and Ty2. |
| scaffold2_15 | 2128 | Homology to *S. cerevisiae BAR1* & *tRNA* & Ty1 LTR. |
| scaffold2_16 | 2076 | Homology to part of *S. cerevisiae* Y' element*. |
| scaffold2_17 | 2062 | Homology to part of *S. cerevisiae SAM* genes. |
| scaffold2_18 | 2024 | Homology to part of *S. cerevisiae* tRNA & Ty. |

* - Location is subtelomeric or telomeric in *S. cerevisiae*.
