# Supplementary figures and images for: High quality de novo sequencing and assembly of the Saccharomyces arboricolus genome
Source: BMC Genomics. 2013 Jan 31;14:69. doi: 10.1186/1471-2164-14-69 (PMC3599269; doi:10.1186/1471-2164-14-69)

Liti et al. **Supplementary Figure S1**

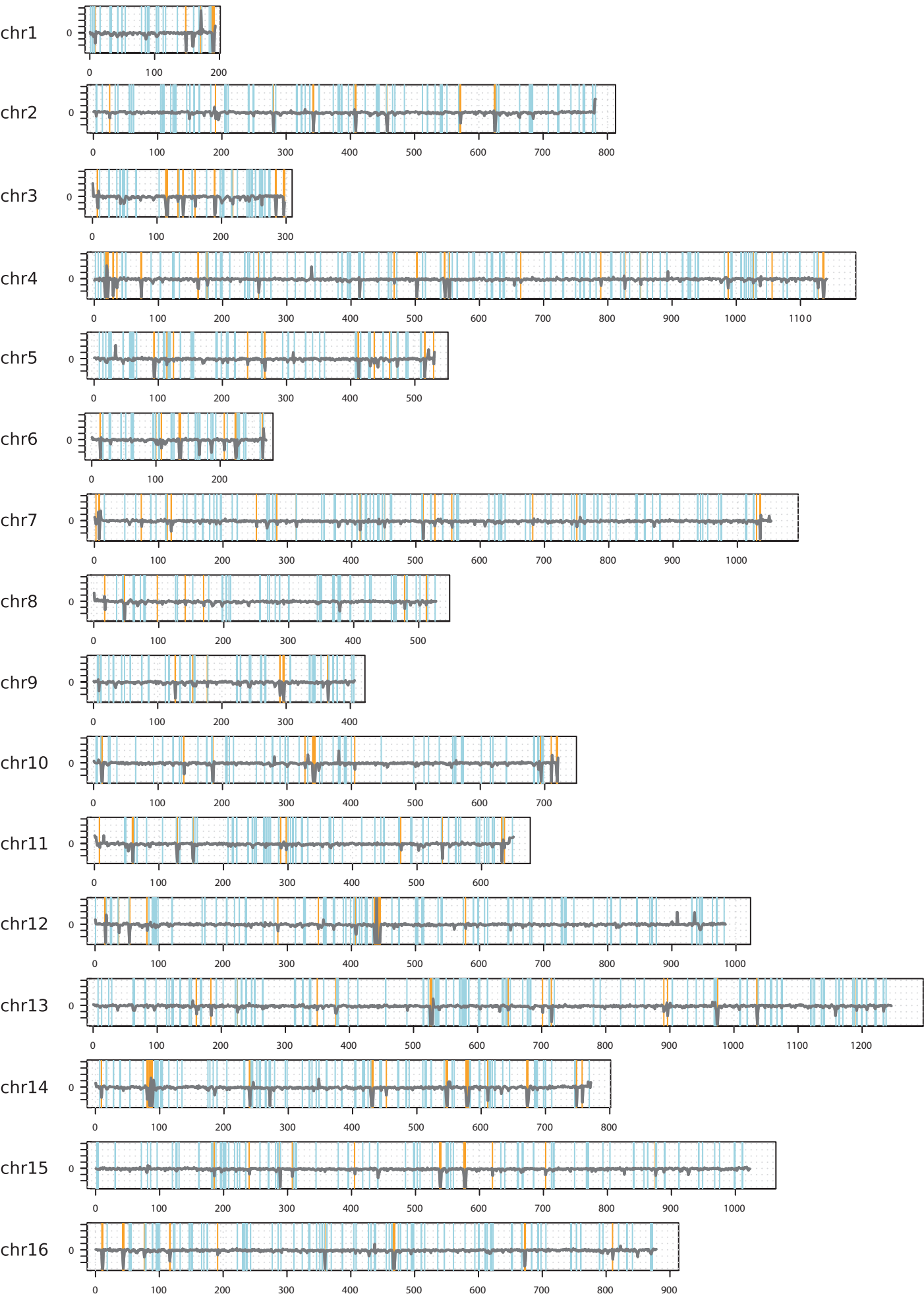

Supplement: Additional file 2: Figure S1 — SOLiD coverage across the 16 chromosomes of S. arboricolus to detect regions of elevated copy number. There is one plot for each chromosome, with the x-axis representing the chromosomal coordinate and the y-axis (on a log2 scale) representing sequence coverage as a measure of copy number (normalized by the genome-wide average). SOLiD reads were mapped to the S. arboricolus assembly using BFAST [ [51]]. Reads with multiple equally good top scoring mapping locations were assigned randomly to one of these. The depth of coverage of reads mapping to the assembly was calculated in windows of size 1 kb along the chromosomes. Each step on the vertical axis corresponds to one unit on the logarithmic scale. Vertical bars in orange mark the locations of gaps in the assembly and bars in blue mark the locations of tandem repeat tracts longer than 30 bp, as predicted by Tandem Repeats Finder [ [52]]. [file 1471-2164-14-69-S2.pdf]

Liti et al. Supplementary Figure S2

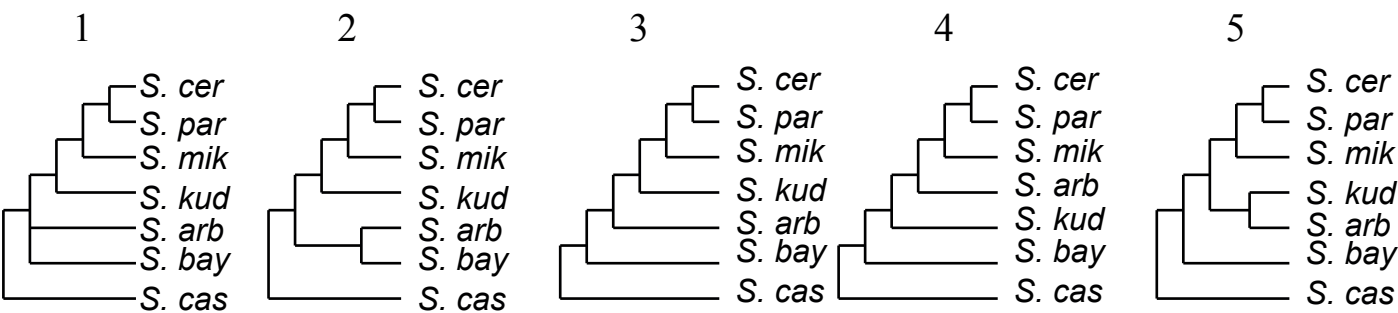

Supplement: Additional file 4: Figure S2 — Five possible placements of S. arboricolus within the sensu stricto complex. We find that the third placement is supported by the data (main manuscript Figure 5). S. cer: S. cerevisiae; S. par: S. paradoxus; S. mik: S. mikatae; S. kud: S. kudriavzevii; S. arb: S. arboricolus; and S. bay: S. bayanus. [file 1471-2164-14-69-S4.pdf]
