# Supplementary material for: High quality de novo sequencing and assembly of the Saccharomyces arboricolus genome
Source: BMC Genomics. 2013 Jan 31;14:69. doi: 10.1186/1471-2164-14-69 (PMC3599269; doi:10.1186/1471-2164-14-69)
Supplement: Additional file 5: Table S2 — Environments used in the phenotyping screen. Classification”carbon utilization” indicates that 2% glucose was substituted with the indicated carbon source, classification “nitrogen utilization” indicates that 0.5% ammonium sulfate was substituted with the indicated nitrogen sources at nitrogen limiting concentrations. In all nitrogen utilization experiments, two consecutive pre-cultures were performed to deplete internal nitrogen storages: the first with nitrogen limiting amounts of ammonium, the second with nitrogen limiting amounts of the indicated nitrogen source. # = pre-cultures were performed in medium similar to the experimental medium to deplete internal storages of the molecule. [file 1471-2164-14-69-S5.docx]

**Supplementary Table S2.** Classification ”carbon utilization” indicates that 2% glucose was substituted with the indicated carbon source, classification “nitrogen utilization” indicates that 0.5% ammonium sulfate was substituted with the indicated nitrogen sources at nitrogen limiting concentrations. In all nitrogen utilization experiments, two consecutive pre-cultures were performed to deplete internal nitrogen storages: the first with nitrogen limiting amounts of ammonium, the second with nitrogen limiting amounts of the indicated nitrogen source. # = pre-cultures were performed in medium similar to the experimental medium to deplete internal storages of the molecule

| Environment | Class |
| --- | --- |
| No stress | - |
| Lactose 2% | Carbon utilization |
| Mannitol 2% | Carbon utilization |
| Melezitose 2% | Carbon utilization |
| Methanol 2% | Carbon utilization |
| Starch 1% | Carbon utilization |
| Trehalose 2% | Carbon utilization |
| Xylitol 2% | Carbon utilization |
| Citric acid 46mg/mL | Environment & Metabolites |
| Citric acid 55mg/mL | Environment & Metabolites |
| Citric acid 62.5mg/mL | Environment & Metabolites |
| DHA 100mM | Environment & Metabolites |
| DHA 200mM | Environment & Metabolites |
| Formaldehyde 1mM | Environment & Metabolites |
| Heat 41°C | Environment & Metabolites |
| Heat 42°C | Environment & Metabolites |
| Heat 43°C | Environment & Metabolites |
| Malic acid 52mg/ml | Environment & Metabolites |
| Malic acid 62.5mg/ml | Environment & Metabolites |
| Methanol 10% | Environment & Metabolites |
| Methanol 2% | Environment & Metabolites |
| Methanol 5% | Environment & Metabolites |
| Methanol 7.5% | Environment & Metabolites |
| Methylglyoxal 2mM | Environment & Metabolites |
| Methylglyoxal 4mM | Environment & Metabolites |
| Oxalic acid 12.5mg/ml | Environment & Metabolites |
| Oxalic acid 8mg/ml | Environment & Metabolites |
| Selenomethionine 0.03mM | Environment & Metabolites |
| Selenomethionine 0.06mM | Environment & Metabolites |
| Synthetic wine must | Environment & Metabolites |
| Tartaric acid 45mg/mL | Environment & Metabolites |
| Tartaric acid 60mg/mL | Environment & Metabolites |
| YPD | Environment & Metabolites |
| Adenine | Nitrogen utilization |
| Alanine | Nitrogen utilization |
| Arginine | Nitrogen utilization |
| Asparagine | Nitrogen utilization |
| Aspartic | Nitrogen utilization |
| Citrulline | Nitrogen utilization |
| Cytidine | Nitrogen utilization |
| Cytosine | Nitrogen utilization |
| GABA | Nitrogen utilization |
| Glutathione | Nitrogen utilization |
| Glycine | Nitrogen utilization |
| Glycine | Nitrogen utilization |
| Guanidine | Nitrogen utilization |
| Histidine | Nitrogen utilization |
| Isoleucine | Nitrogen utilization |
| Leucine | Nitrogen utilization |
| Low | Nitrogen utilization |
| Methionine | Nitrogen utilization |
| Ornithine | Nitrogen utilization |
| Phenylalanine | Nitrogen utilization |
| Proline | Nitrogen utilization |
| Serine | Nitrogen utilization |
| Threonine | Nitrogen utilization |
| Tryptophane | Nitrogen utilization |
| Tyrosine | Nitrogen utilization |
| Urea | Nitrogen utilization |
| Valine | Nitrogen utilization |
| Adenine (-) | Nutrient requirements |
| Aminoacids (-)# | Nutrient requirements |
| Biotin (-) | Nutrient requirements |
| Biotin (-)# | Nutrient requirements |
| Boron (-)# | Nutrient requirements |
| Ca (-) | Nutrient requirements |
| Ca (-)# | Nutrient requirements |
| Cu (-) | Nutrient requirements |
| Fe (-)# | Nutrient requirements |
| Inositol (-)# | Nutrient requirements |
| Inositol (-) | Nutrient requirements |
| Leucine (-)# | Nutrient requirements |
| Lysine (-) | Nutrient requirements |
| Methionine (-) | Nutrient requirements |
| Mg (-)# | Nutrient requirements |
| Mg (-) | Nutrient requirements |
| Mn (-)# | Nutrient requirements |
| Mo (-)# | Nutrient requirements |
| Na (-)# | Nutrient requirements |
| Nicotinamide (-) | Nutrient requirements |
| Nicotinamide (-)# | Nutrient requirements |
| PABA (-) | Nutrient requirements |
| PABA (-)# | Nutrient requirements |
| Pantothenate (-) | Nutrient requirements |
| Pantothenate (-)# | Nutrient requirements |
| Phosphate (-)# | Nutrient requirements |
| Phosphate (-) | Nutrient requirements |
| Pyridoxin (-)# | Nutrient requirements |
| Riboflavin (-)# | Nutrient requirements |
| Thiamine (-)# | Nutrient requirements |
| Thiamine (-) | Nutrient requirements |
| Tryptophan (-)# | Nutrient requirements |
| Uracil (-) | Nutrient requirements |
| Zn (-)# | Nutrient requirements |
| 1,10 Phenanthroline 2µM | Toxins |
| 1,10 phenantroline 4µM | Toxins |
| 6-azauracil 200µg/mL | Toxins |
| 6-azauracil 300µg/mL | Toxins |
| 6-azauracil 500µg/mL | Toxins |
| Canavanine 100µg/mL | Toxins |
| DMSO 0.25% | Toxins |
| DMSO 2% | Toxins |
| DMSO 4% | Toxins |
| DMSO 8% | Toxins |
| DTT 1.6mM | Toxins |
| DTT 1.8mM | Toxins |
| Geneticin 2mM | Toxins |
| Geneticin 3mM | Toxins |
| Geneticin 4mM | Toxins |
| Hog1 inhibitor TA47:2 2µM | Toxins |
| Hog1p inhibitor TA47:2 8uM | Toxins |
| Hygromycin 0.6mg/mL | Toxins |
| Hygromycin B 1.25mg/ml | Toxins |
| Mercaptoethanol 15mM | Toxins |
| Mercaptoethanol 30mM | Toxins |
| Noursethricine 10µg/mL | Toxins |
| Noursethricine 100µg/mL | Toxins |
| Noursethricine 5µg/ml | Toxins |
| Staurosporine 10ug/mL | Toxins |
| Staurosporine 1ug/mL | Toxins |
| Thiabendazole 25µg/ml | Toxins |
| Thiabendazole 50µg/ml | Toxins |
